# Risk factors for re-amputation and major amputation following diabetic foot amputation: a clinical and socioeconomic perspective

**DOI:** 10.1186/s12893-026-03777-4

**Published:** 2026-04-25

**Authors:** Yeongchan Kim, Hongki Park

**Affiliations:** Department of Orthopedic Surgery, Gachon University Gil Medical Center, 783 Namdong-daero, Namdong-gu, Incheon, Republic of Korea

**Keywords:** Diabetic foot, Amputation, Risk factors, Social determinants of health, Cohort studies

## Abstract

**Background:**

Diabetic foot amputation (DFA) is a severe complication of diabetes mellitus, and many patients require subsequent procedures because of recurrence or progression of ischemia. Although the clinical predictors of amputation have been extensively studied, the influence of social factors remains underexplored in Asian populations.

**Methods:**

This retrospective cohort included 155 patients with diabetic foot ulcers who presented between 2024 and 2025 and had undergone surgical amputation. Immediate in-hospital revision procedures were excluded, as these were considered part of the initial surgical episode rather than true re-amputation. Amputations were categorized as minor or major. Group comparisons were performed using t-tests or chi-square tests, and variables with *p* < 0.10 in univariate analyses were entered into multivariate logistic regression to identify independent predictors of re-amputation and major amputation. Kaplan–Meier survival analysis and Cox proportional hazards regression were additionally performed to evaluate time to re-amputation.

**Results:**

The re-amputation rate was 52.3%, and major amputations occurred in 14.2%. On multivariate analysis, higher BMI and marital status predicted re-amputation. For major amputation, insulin or combined therapy, and marital status were predictors. Elevated CRP and WBC levels were associated with major amputation in univariate analyses but lost significance after adjustment.

**Conclusions:**

Both clinical and social determinants influenced adverse outcomes following DFA, with marital status consistently predicting both re-amputation and major amputation.

**Supplementary Information:**

The online version contains supplementary material available at 10.1186/s12893-026-03777-4.

## Introduction

Diabetes mellitus is a major cause of non-traumatic lower extremity amputation, representing one of the most severe complications of the disease and a substantial public health burden [1, 2]. Although diabetic foot ulcer (DFU) is the primary cause of limb loss, amputation often represents the beginning of a prolonged treatment journey rather than its end. Many patients require subsequent procedures because of recurrent ulceration, infection, or progression of ischemia, and these re-amputations are strongly linked to impaired functional outcomes, reduced quality of life, and increased mortality [3–5].

Recent studies have investigated the risk factors for adverse outcomes following diabetic foot amputation. Clinical predictors such as bone involvement, hindfoot ulcer location, chronic renal failure, dialysis, anemia, and poor glycemic control have consistently been associated with the risk of major amputation [6–8]. A previous study reported that bone invasion, dialysis, gastrointestinal disorders, and low hemoglobin levels were independent predictors of major amputation in hospitalized patients with diabetic foot ulcers (DFUs) [6]. Furthermore, repeated lesions after forefoot amputation have been shown to occur frequently, often leading to contralateral disease or progression to proximal amputation [3, 9].

Previous studies have largely emphasized the clinical determinants of amputation, including vascular status and systemic comorbidities [5–8]. More recently, evidence has emerged regarding the influence of socioeconomic and psychosocial factors, such as income, insurance coverage, and living status, on outcomes [10, 11]. In addition to these patient-level factors, emerging research has identified structural determinants such as healthcare accessibility, regional deprivation, and educational level as independent predictors of diabetic foot outcomes [12]. Furthermore, several reviews and recent machine-learning studies have explored prediction models for amputation, emphasizing the need to integrate both clinical and social determinants to improve early risk stratification [13, 14]. However, few studies have examined whether these socioeconomic and structural determinants influence the risk of re-amputation or the progression from minor to major amputations, particularly in Asian populations.

Accordingly, this study’s aim was threefold: (1) to identify factors associated with re-amputation among patients who underwent diabetic foot amputation; (2) to evaluate the clinical and social determinants of major amputation; and (3) to further characterize the predictors of amputation levels, ranging from toe to above-knee amputation. By addressing these questions, this study aimed to improve risk stratification and develop individualized strategies for prevention and management.

## Materials and methods

This study retrospectively reviewed 292 patients with foot ulcers who presented to a tertiary referral center between August 2024 and August 2025. Only adult patients aged ≥ 18 years were included in the study. Among them, 155 patients who had undergone surgical amputation, defined as a treatment beyond simple debridement, were included in the final analysis. The exclusion criteria included traumatic amputations, amputations due to non-diabetic causes (such as tumors or congenital deformities), and cases with insufficient clinical data.

The evaluated variables included potential risk factors for re-amputation and major amputation, such as gender, age, body mass index (BMI), diabetes duration, comorbidities (hypertension, chronic kidney disease, coronary artery disease), laboratory parameters (white blood cell count, C-reactive protein, and HbA1c), lesion characteristics [including ulcer depth according to the Texas classification (simplified as superficial [Grade 1–2] and deep [Grade 3]), location, and ankle–brachial index (ABI)], and socioeconomic factors (marital status, living status). Renal function was additionally evaluated using the estimated glomerular filtration rate (eGFR), calculated from serum creatinine values using the Chronic Kidney Disease Epidemiology Collaboration (CKD-EPI) equation. Dialysis was defined as either hemodialysis or peritoneal dialysis. Bone invasion was defined as radiologic or intraoperative evidence of osteomyelitis.

Amputations were categorized as minor (toe, ray, transmetatarsal, or Chopart) or major (Syme, below-knee, or above-knee). Re-amputation was defined as any subsequent amputation performed after hospital discharge or during a later admission, regardless of whether it occurred on the same limb or the contralateral limb. This included cases in which patients had undergone a previous amputation at another institution and subsequently required additional amputation at our hospital. This definition was used to distinguish true re-amputation events from staged procedures performed during the index hospitalization. In cases of bilateral amputations, the limb with the higher amputation level was used for analysis. Amputations performed within the same hospitalization period, such as progression from toe to ray amputation within a few days due to uncontrolled infection, were not considered re-amputations, as these were regarded as part of the index treatment. The primary outcomes were incidence of re-amputation, time interval to re-amputation, and occurrence of additional amputations at other anatomical sites. Follow-up for the assessment of re-amputation and major amputation outcomes continued until August 31, 2025.

All statistical analyses were performed using SPSS software (version 23.0; IBM Corp., Armonk, NY, USA). Continuous variables were expressed as mean ± standard deviation (SD). For two-group comparisons, Student’s t-test or the Mann–Whitney U test was used as appropriate. For comparisons across multiple amputation levels, the Kruskal–Wallis test was applied because of non-normal distribution and small subgroup sizes. Categorical variables were compared using the χ² test (with Yates’ correction when appropriate) or Fisher’s exact test when expected cell counts were < 5. Variables with *p* < 0.10 in univariate analysis were entered into a multivariate logistic regression model to identify independent predictors of re-amputation and major amputation. To account for differences in follow-up duration among patients, Kaplan–Meier survival analysis was performed to evaluate time to re-amputation. In addition, Cox proportional hazards regression analysis was conducted to assess predictors of time to re-amputation. Because the number of major amputation events was relatively small, Firth’s penalized logistic regression analysis was additionally performed to reduce potential small-sample bias. Statistical significance was set at a two-tailed p-value < 0.05. Missing values were excluded from the corresponding analyses, and no statistical imputation was performed.

This study was approved by the Institutional Review Board (approval no. GCIRB2025-294). The requirement for informed consent was waived due to the retrospective design.

Artificial intelligence–based language models (ChatGPT, OpenAI) were used solely for language refinement. All study design, statistical analyses, interpretation, and conclusions were independently performed by the authors.

## Results

In total, 155 patients who underwent surgical amputation for diabetic foot lesions were included in the final analysis. The overall re-amputation rate was 52.3% (81/155), and major amputations accounted for 14.2% (22/155). The median interval to re-amputation was 203 days (IQR, 70–748 days). The median follow-up time was 527 days (IQR, 237–1114 days). Baseline characteristics and group comparisons are summarized in Tables 1, 2, 3 and 4.

**Table 1:** Demographics and univariate analysis of 155 patients with and without re-amputation

	Total	Absence of Re-amputation		Re-amputation	*P* value
	(*N* = 155, %)	(*N* = 74, %)	(*N* = 81, %)		
Gender					0.62
Male	120 (77.4)	56 (75.7)	64 (79.0)		
Female	35 (22.6)	18 (24.3)	17 (21.0)		
Age (years, mean ± SD)	63.43 ± 11.69	64.89 ± 12.16	62.09 ± 11.16		0.14
Marital status					**< 0.01**
Married	91 (58.7)	53 (71.6)	38 (46.9)		
Single	24 (15.5)	11 (14.9)	13 (16.0)		
Divorced/Widowed	40 (25.8)	10 (13.5)	30 (37.0)		
Living status					**0.01**
Living alone	59 (38.1)	20 (27.0)	39 (48.1)		
Living with family	96 (61.9)	54 (73.0)	42 (51.9)		
Smoking					0.53
Yes	64 (41.3)	33 (44.6)	31 (38.3)		
No	91 (58.7)	41 (55.4)	50 (61.7)		
Alcohol consumption					0.28
Yes	49 (31.6)	27 (36.5)	22 (27.2)		
No	106 (68.4)	47 (63.5)	59 (72.8)		
Hypertension					0.05
Yes	99 (63.9)	41 (55.4)	58 (71.6)		
No	56 (36.1)	33 (44.6)	23 (28.4)		
CKD					0.26
Yes	40 (25.8)	16 (21.6)	24 (29.6)		
No	115 (74.2)	58 (78.4)	57 (70.4)		
Dialysis					0.31
Yes	19 (12.3)	7 (9.5)	12 (14.8)		
No	136 (87.7)	67 (90.5)	69 (85.2)		
CAD					0.42
Yes	27 (17.4)	11 (14.9)	16 (19.8)		
No	128 (82.6)	63 (85.1)	65 (80.2)		
DM treatment					**0.04**
Oral agents	121 (78.1)	63 (85.1)	58 (71.6)		
Insulin or combined	34 (21.9)	11 (14.9)	23 (28.4)		
Texas classification (Simplified 2-tier model)					0.35
Superficial (Grade1–2)	11 (7.1)	7 (9.5)	4 (4.9)		
Deep (Grade 3)	144 (92.9)	67 (90.5)	77 (95.1)		
BMI (kg/m² ± SD)	24.13 ± 4.09	23.38 ± 4.66	24.82 ± 3.39		**0.03**
eGFR (mL/min/1.73 m²)	67.72 ± 31.17	70.86 ± 30.24	64.86 ± 31.91		0.23
ABI	1.03 ± 0.29	1.00 ± 0.31	1.07 ± 0.26		0.13
HbA1c (%)	8.75 ± 2.33	8.63 ± 2.56	8.86 ± 2.11		0.55
CRP (mg/L)	8.49 ± 9.48	7.74 ± 8.51	9.18 ± 10.29		0.34
WBC (10³/µL)	12.08 ± 7.37	12.02 ± 7.01	12.14 ± 7.72		0.92

Data are presented as mean ± SD or n (%). Bold values indicate *p* < 0.05

Combined indicates patients receiving both oral hypoglycemic agents and insulin therapy

**Table 2:** Demographics and univariate analysis of 155 patients with and without major amputation

	Total	Minor amputation	Major amputation	*P* value
	(*N* = 155, %)	(*N* = 133, %)	(*N* = 22, %)	
Gender				0.17
Male	120 (77.4)	100 (75.2)	20 (90.9)	
Female	35 (22.6)	33 (24.8)	2 (9.1)	
Age (years, mean ± SD)	63.43 ± 11.69	63.70 ± 12.02	61.77 ± 9.60	0.41
Marital status				**0.02**
Married	91 (58.7)	84 (63.2)	7 (31.8)	
Single	24 (15.5)	18 (13.5)	6 (27.3)	
Divorced/Widowed	40 (25.8)	31 (23.3)	9 (40.9)	
Living status				**0.03**
Living alone	59 (38.1)	46 (34.6)	13 (59.1)	
Living with family	96 (61.9)	87 (65.4)	9 (40.9)	
Smoking				0.37
Yes	64 (41.3)	53 (39.8)	11 (50.0)	
No	91 (58.7)	80 (60.2)	11 (50.0)	
Alcohol consumption				0.64
Yes	49 (31.6)	43 (32.3)	6 (27.3)	
No	106 (68.4)	90 (67.7)	16 (72.7)	
Hypertension				1.00
Yes	99 (63.9)	85 (63.9)	14 (63.6)	
No	56 (36.1)	48 (36.1)	8 (36.4)	
CKD				0.72
Yes	40 (25.8)	35 (26.3)	5 (22.7)	
No	115 (74.2)	98 (73.7)	17 (77.3)	
Dialysis				0.89
Yes	19 (12.3)	17 (12.8)	2 (9.1)	
No	136 (87.7)	116 (87.2)	20 (90.9)	
CAD				0.19
Yes	27 (17.4)	21 (15.8)	6 (27.3)	
No	128 (82.6)	112 (84.2)	16 (72.7)	
DM treatment				**0.02**
Oral agents	121 (78.1)	108 (81.2)	13 (59.1)	
Insulin or combined	34 (21.9)	25 (18.8)	9 (40.9)	
Texas classification (Simplified 2-tier model)				1.00
Superficial (Grade1–2)	11 (7.1)	10 (7.5)	1 (4.5)	
Deep (Grade 3)	144 (92.9)	123 (92.5)	21 (95.5)	
BMI (kg/m² ± SD)	24.13 ± 4.09	24.20 ± 4.14	23.74 ± 3.85	0.38
eGFR (mL/min/1.73 m²)	67.72 ± 31.17	65.86 ± 30.60	78.99 ± 32.88	0.07
ABI	1.03 ± 0.29	1.04 ± 0.29	0.95 ± 0.32	0.07
HbA1c (%)	8.75 ± 2.33	8.70 ± 2.30	9.05 ± 2.57	0.78
CRP (mg/L)	8.49 ± 9.48	7.54 ± 8.77	14.22 ± 11.63	**< 0.01**
WBC (10³/µL)	12.08 ± 7.37	11.29 ± 6.50	16.87 ± 10.21	**< 0.01**

Data are presented as mean ± SD or n (%). Bold values indicate *p* < 0.05

Combined indicates patients receiving both oral hypoglycemic agents and insulin therapy

**Table 3:** Multivariate logistic regression analysis of demographic and clinical factors associated with re-amputation and major amputation

Variable	OR (Re-amputation)	95% CI	*p*-value	OR (Major amputation)	95% CI	*p*-value
Demographic factors						
Marital status¹	1.24	0.44–3.54	0.69	11.17	1.66–75.07	**0.01**
Marital status²	3.38	1.12–10.22	**0.03**	10.04	1.19–84.46	**0.03**
Living status³	0.81	0.31–2.11	0.67	2.28	0.41–12.81	0.35
Clinical factors						
BMI (kg/m²)	1.10	1.00–1.20	**0.04**	-	-	-
DM treatment⁴	2.17	0.95–5.12	0.08	4.26	1.07–16.95	**0.04**
Hypertension⁵	1.84	0.90–3.77	0.09	-	-	**-**
Laboratory factors						
ABI⁶	-	-	-	0.11	0.01–1.00	0.05
CRP (mg/L)	-	-	-	1.04	0.96–1.12	0.39
WBC (10³/µL)	-	-	-	1.06	0.97–1.15	0.23

Variables with *p* < 0.10 in univariate analysis were included in the multivariate logistic regression model

Bold values indicate statistical significance (*p* < 0.05). OR, odds ratio; CI, confidence interval

¹ Never married vs. married; ² Divorced/Widowed vs. married; ³ Living alone vs. living with family; ⁴ Insulin or combined therapy vs. oral agents; ⁵ Hypertension vs. no hypertension; ⁶ ABI indicates ankle-brachial index; per 1-unit increase

Reference categories for the categorical variables were married, living with family, oral agents, and no hypertension

**Table 4:** Demographics, clinical characteristics, and laboratory findings of patients according to amputation level

	Total	Toe	Ray	Transmetatarsal	Chopart	Syme	BK	AK	*P* value
	(*N* = 155, %)	(*N* = 41, %)	(*N* = 63, %)	(*N* = 22, %)	(*N* = 7, %)	(*N* = 4, %)	(*N* = 15, %)	(*N* = 3, %)	
Gender									0.30
Male	120 (77.4)	27 (65.9)	50 (79.4)	18 (81.8)	5 (71.4)	4 (100.0)	14 (93.3)	2 (66.7)	
Female	35 (22.6)	14 (34.1)	13 (20.6)	4 (18.2)	2 (28.6)	0 (0.0)	1 (6.7)	1 (33.3)	
Age (years ± SD)	63.4 ± 11.7	66.6 ± 12.2	62.1 ± 12.0	61.5 ± 11.4	68.7 ± 10.9	60.8 ± 8.1	60.2 ± 9.3	71.0 ± 10.6	0.21
Marital status									0.41
Married	91 (58.7)	26 (63.4)	39 (61.9)	14 (63.6)	5 (71.4)	0 (0.0)	6 (40.0)	1 (33.3)	
Single	24 (15.5)	7 (17.1)	8 (12.7)	3 (13.6)	0 (0.0)	2 (50.0)	3 (20.0)	1 (33.3)	
Divorced/Widowed	40 (25.8)	8 (19.5)	16 (25.4)	5 (22.7)	2 (28.6)	2 (50.0)	6 (40.0)	1 (33.3)	
Living status									0.43
Living alone	59 (38.1)	13 (31.7)	22 (34.9)	8 (36.4)	3 (42.9)	3 (75.0)	8 (53.3)	2 (66.7)	
Living with family	96 (61.9)	28 (68.3)	41 (65.1)	14 (63.6)	4 (57.1)	1 (25.0)	7 (46.7)	1 (33.3)	
Smoking									0.55
Yes	64 (41.3)	17 (41.5)	25 (39.7)	9 (40.9)	2 (28.6)	2 (50.0)	9 (60.0)	0 (0.0)	
No	91 (58.7)	24 (58.5)	38 (60.3)	13 (59.1)	5 (71.4)	2 (50.0)	6 (40.0)	3 (100.0)	
Alcohol consumption									0.43
Yes	49 (31.6)	18 (43.9)	17 (27.0)	7 (31.8)	1 (14.3)	1 (25.0)	5 (33.3)	0 (0.0)	
No	106 (68.4)	23 (56.1)	46 (73.0)	15 (68.2)	6 (85.7)	3 (75.0)	10 (66.7)	3 (100.0)	
Hypertension									0.52
Yes	99 (63.9)	25 (61.0)	38 (60.3)	18 (81.8)	4 (57.1)	3 (75.0)	10 (66.7)	1 (33.3)	
No	56 (36.1)	16 (39.0)	25 (39.7)	4 (18.2)	3 (42.9)	1 (25.0)	5 (33.3)	2 (66.7)	
CKD									0.35
Yes	40 (25.8)	8 (19.5)	19 (30.2)	8 (36.4)	0 (0.0)	0 (0.0)	4 (26.7)	1 (33.3)	
No	115 (74.2)	33 (80.5)	44 (69.8)	14 (63.6)	7 (100.0)	4 (100.0)	11 (73.3)	2 (66.7)	
Dialysis									0.63
Yes	19 (12.3)	3 (7.3)	10 (15.9)	4 (18.2)	0 (0.0)	0 (0.0)	2 (13.3)	0 (0.0)	
No	136 (87.7)	38 (92.7)	53 (84.1)	18 (81.8)	7 (100.0)	4 (100.0)	13 (86.7)	3 (100.0)	
CAD									**0.01**
Yes	27 (17.4)	4 (9.8)	6 (9.5)	9 (40.9)	2 (28.6)	1 (25.0)	5 (33.3)	0 (0.0)	
No	128 (82.6)	37 (90.2)	57 (90.5)	13 (59.1)	5 (71.4)	3 (75.0)	10 (66.7)	3 (100.0)	
DM treatment									0.11
Oral agents	121 (78.1)	33 (80.5)	52 (82.5)	17 (77.3)	6 (85.7)	1 (25.0)	9 (60.0)	3 (100.0)	
Insulin or combined	34 (21.9)	8 (19.5)	11 (17.5)	5 (22.7)	1 (14.3)	3 (75.0)	6 (40.0)	0 (0.0)	
Texas classification (Simplified 2-tier model)									0.39
Superficial (Grade1–2)	11 (7.1)	6 (14.6)	4 (6.3)	0 (0.0)	0 (0.0)	0 (0.0)	1 (6.7)	0 (0.0)	
Deep (Grade 3)	144 (92.9)	35 (85.4)	59 (93.7)	22 (100.0)	7 (100.0)	4 (100.0)	14 (93.3)	3 (100.0)	
BMI (kg/m² ± SD)	24.1 ± 4.1	24.0 ± 4.5	24.4 ± 4.0	24.9 ± 3.9	21.2 ± 2.8	23.8 ± 2.6	24.2 ± 4.1	21.1 ± 4.2	0.38
ABI	1.0 ± 0.3	1.1 ± 0.3	1.1 ± 0.3	1.0 ± 0.3	0.9 ± 0.3	0.9 ± 0.3	1.1 ± 0.2	0.5 ± 0.4	0.07
HbA1c (%)	8.7 ± 2.3	8.5 ± 2.1	8.8 ± 2.5	8.3 ± 1.9	9.7 ± 3.0	8.9 ± 3.2	8.9 ± 2.4	9.9 ± 3.6	0.93
CRP (mg/L)	8.5 ± 9.5	4.4 ± 5.5	8.1 ± 7.7	8.5 ± 12.3	17.9 ± 12.2	11.9 ± 13.7	14.3 ± 12.3	17.0 ± 7.7	**< 0.01**
WBC (10³/µL)	12.1 ± 7.4	9.2 ± 4.4	11.7 ± 6.7	11.4 ± 6.9	19.5 ± 7.6	14.1 ± 6.8	17.5 ± 11.4	17.5 ± 10.1	**< 0.01**

Data are presented as mean ± standard deviation or number (%)

*CRP* C-reactive protein, *WBC* White blood cell count, *BMI* Body mass index, *ABI* Ankle-brachial index, *CAD* Coronary artery disease, *CKD* Chronic kidney disease, *BK* Below-knee amputation, *AK* Above-knee amputation. Bold values indicate *p* < 0.05

### Re-amputation

In the univariate analysis (Table 1), re-amputation was significantly associated with marital status (*p* < 0.01), living status (*p* = 0.01), and BMI (*p* = 0.03) and showed a trend toward significance for hypertension (*p* = 0.05).

In the multivariate model, marital status (divorced/widowed vs. married; OR 3.38, 95% CI 1.12–10.22, *p* = 0.03) and BMI (OR 1.10, 95% CI 1.00–1.20, *p* = 0.04) remained as independent predictors of re-amputation, while living status and hypertension lost statistical significance in the multivariable model.

### Major amputation

In the univariate analysis (Table 2), major amputation was significantly associated with marital status (*p* = 0.02), living status (*p* = 0.03), diabetes treatment (*p* = 0.02), C-reactive protein (CRP) (*p* < 0.01), and WBC count (*p* < 0.01).

In the multivariate analysis, marital status (never married vs. married; OR 11.17, 95% CI 1.66–75.07, *p* = 0.01; divorced/widowed vs. married; OR 10.04, 95% CI 1.19–84.46, *p* = 0.03) and diabetes treatment (insulin or combined therapy vs. oral agents; OR 4.26, 95% CI 1.07–16.95, *p* = 0.04) remained as independent risk factors for major amputation, whereas CRP and WBC lost statistical significance. To address the potential small-sample bias resulting from the limited number of major amputation events relative to the number of variables included in the model, an additional Firth penalized logistic regression analysis was performed (Supplementary Table S1). In this analysis, never-married status (OR 8.79, 95% CI 1.52–50.74, *p* = 0.02) and divorced/widowed status (OR 7.82, 95% CI 1.12–54.76, *p* = 0.04) remained significantly associated with major amputation. Insulin or combined therapy (OR 3.62, 95% CI 0.99–13.24, *p* = 0.05) and ABI (OR 0.14, 95% CI 0.02–1.08, *p* = 0.06) showed borderline significance, whereas CRP and WBC remained non-significant. Overall, the direction and magnitude of the associations were consistent with those observed in the conventional multivariable logistic regression model.

### Amputation level analysis

When analyzed according to the amputation level (Table 4), significant differences were observed in CRP (*p* < 0.01), WBC (*p* < 0.01), and the prevalence of coronary artery disease (CAD) (*p* = 0.01).

### Kaplan–meier analysis

**Fig. 1:**
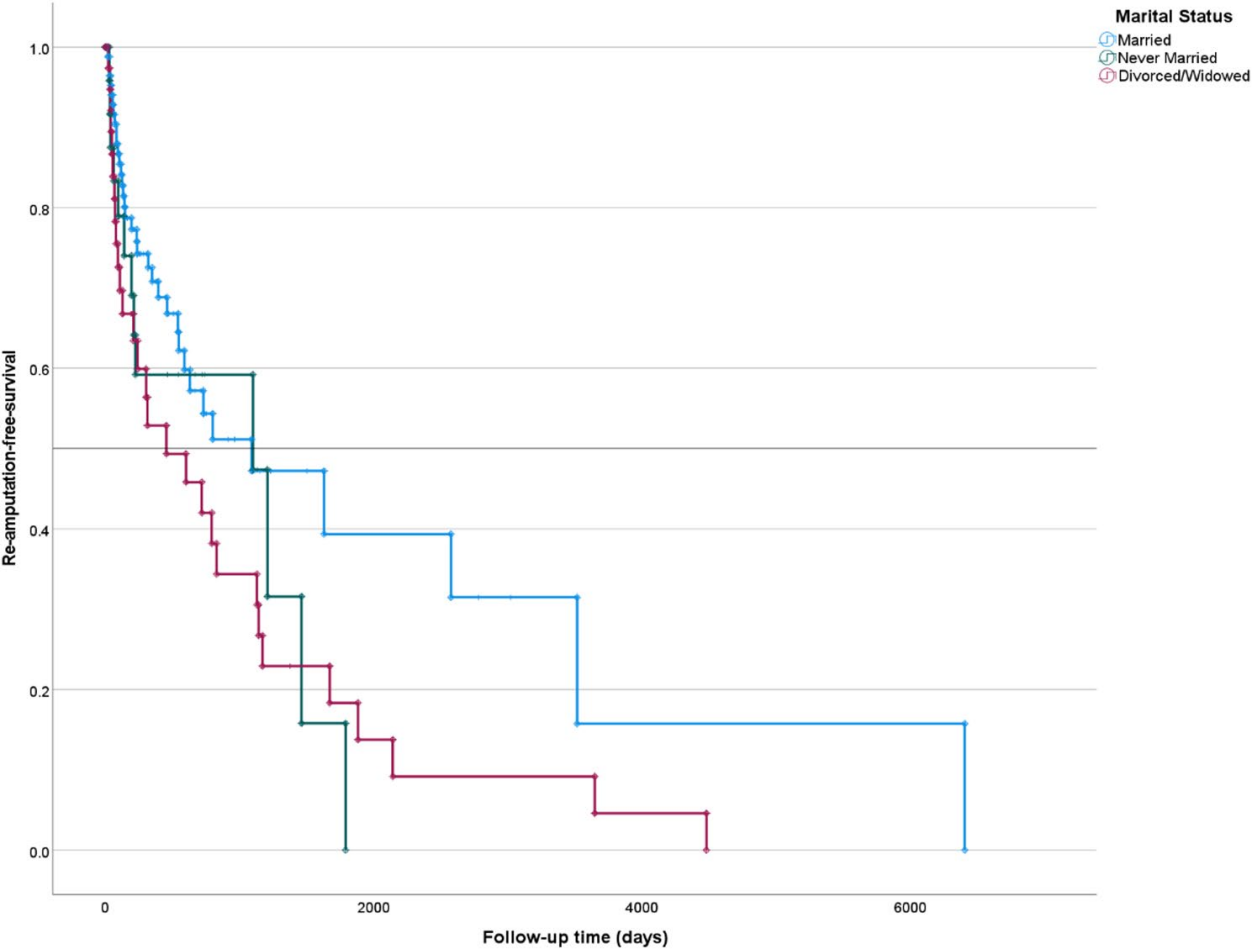
Kaplan–Meier curve for re-amputation-free survival according to marital status. Divorced or widowed patients demonstrated a significantly higher risk of re-amputation compared with married patients (log-rank *p* = 0.02)

To account for differences in follow-up duration among patients, Kaplan–Meier survival analysis was performed to evaluate time to re-amputation. The overall median re-amputation-free survival was 801 days (95% CI 382–1219). When stratified by marital status, divorced or widowed patients demonstrated a shorter re-amputation-free survival (median 456 days) compared with married (1092 days) and single patients (1101 days). The difference between groups was significant according to the log-rank test (*p* = 0.02). The Kaplan–Meier curve illustrating re-amputation-free survival is shown in Fig. 1.

### Cox proportional hazards regression

Cox proportional hazards regression analysis was performed to evaluate predictors of time to re-amputation, adjusting for marital status, BMI, diabetes treatment, and hypertension (Table 5). In the multivariable model, divorced or widowed marital status was independently associated with an increased hazard of re-amputation compared with married patients (HR 1.72, 95% CI 1.03–2.87; *p* = 0.04). In contrast, single marital status was not significantly associated with re-amputation risk (HR 1.47, 95% CI 0.77–2.80; *p* = 0.24). Other clinical factors, including BMI, diabetes treatment modality, and hypertension, were not independently associated with the risk of re-amputation.

**Table 5:** Multivariable Cox proportional hazards regression analysis identifying predictors of time to re-amputation following diabetic foot amputation

Variable	HR (Re-amputation)	95% CI	*p*-value
Marital status¹	1.47	0.77–2.80	0.24
Marital status²	1.72	1.03–2.87	**0.04**
BMI (kg/m²)	1.01	0.96–1.08	0.64
DM treatment³	1.32	0.79–2.18	0.29
Hypertension⁴	1.20	0.71–2.03	0.49

¹ Never married vs. married; ² Divorced/Widowed vs. married; ³ Insulin or combined therapy vs. oral agents; ⁴ Hypertension vs. no hypertension

Reference categories for the categorical variables were Married, Oral agents, and No hypertension

## Discussion

The global incidence of diabetes-related lower extremity amputation has remained persistently high between 2010 and 2020 [1, 15]. During the long-term management of DFUs, recurrence, readmission, and cumulative healthcare expenditures continue to impose substantial burdens on patients and healthcare systems [2, 3]. In the present single-center cohort, higher BMI and absence of a spouse were identified as independent predictors of re-amputation, whereas insulin or combined therapy and absence of a spouse were independently associated with major amputation. Furthermore, higher amputation levels were accompanied by elevated CRP levels and WBC counts and an increased prevalence of CAD.

In this study, increased BMI was identified as an independent risk factor for re-amputation. This finding may be explained by impaired granulation tissue formation, delayed fibroblast migration, and reduced collagen synthesis caused by hyperglycemia and metabolic overload, as well as by persistent chronic inflammation and microvascular dysfunction that collectively delay wound healing and increase re-amputation risk [16]. Furthermore, an extreme BMI (underweight and overweight) can adversely affect the healing and prognosis of DFUs. Previous studies have reported that low BMI or malnutrition is associated with delayed DFU healing and higher amputation risk [17], whereas high BMI contributes to localized ischemia and microtrauma through increased plantar pressure and shear stress, thereby predisposing patients to recurrent lesions and re-amputation [18–20].

Malnutrition has been increasingly recognized as an important contributor to poor outcomes in patients with diabetic foot disease [21]. Previous studies have reported a high prevalence of malnutrition among these patients, with poor nutritional status being associated with impaired wound healing and an increased risk of amputation [21, 22]. Objective nutritional markers such as the Prognostic Nutritional Index (PNI) have been proposed as useful predictors of limb loss, as malnutrition directly impairs immune function, collagen synthesis, and tissue repair [22]. In addition, emerging evidence highlights that sarcopenia, dynapenia, and frailty further exacerbate these outcomes [23]. Reduced muscle mass and strength are closely linked to metabolic dysfunction and systemic inflammation and may also impair mobility and alter gait mechanics, thereby increasing the likelihood of ulcer progression and subsequent major amputation [23].

Previous studies have highlighted the influences of socioeconomic position and access to care on diabetic foot outcomes. Large population-based studies from the United Kingdom and France have shown that individuals living in socially deprived areas have significantly higher risks of DFUs and lower-limb amputations [11, 24]. Similarly, a nationwide Korean cohort demonstrated that low-income and medical aid beneficiaries had more than twice the risk of amputation and mortality than higher-income groups [10]. However, the association between marital status and diabetic foot amputation has rarely been addressed. A recent Swedish national cohort including 66,569 individuals with newly diagnosed diabetes found that divorced individuals had a significantly higher risk of lower-limb amputation compared with those who were married (adjusted HR 1.67, 95% CI 1.07–2.60), whereas the elevated risk among widowed individuals became non-significant after age adjustment [25]. In our cohort, the absence of a spouse (divorced or widowed) was independently associated with both re-amputation and major amputation, whereas living alone was associated with adverse outcomes only in univariate analyses. These findings suggest that beyond socioeconomic deprivation, the lack of emotional and familial support may play a critical role in postoperative management and risk of recurrence after diabetic foot amputation. In addition, diabetes-related cognitive impairment may further contribute to this association. Chronic hyperglycemia and long-standing diabetes have been linked to cognitive decline, which may impair adherence to complex limb-salvage treatment protocols. In patients with diabetic foot ulcers, effective management requires meticulous wound care, strict glycemic control, and regular clinical follow-up. In the absence of adequate caregiver or spousal support, these cognitive limitations may further compromise treatment adherence and increase the risk of major amputation [26].

In this study, elevated CRP and WBC levels, as well as insulin or combined therapy, were significantly associated with major amputation. Numerous studies have reported associations between inflammatory markers (e.g., CRP and WBC) and the risk of amputation in DFU, and composite indices such as the CRP-to-albumin ratio (CAR) have shown added value in reflecting infection severity and clinical outcomes in DFU [27–29]. A previous study reported that hospitalized patients with DFUs who underwent major amputation exhibited markedly higher WBC count, erythrocyte sedimentation rate (ESR), and CRP levels, and that bony invasion, dialysis, hindfoot ulcer location, low hemoglobin, and elevated fasting glucose remained independent predictors in multivariate analysis [6]. Another study found that elevated WBC count and ESR were associated with major amputation in patients with hindfoot ulcers [7], while one study demonstrated that inflammatory markers (CRP and WBC) were significantly correlated with early major amputation after initial forefoot amputation, with renal dysfunction and dialysis being identified as independent risk factors [8]. In this study, although CRP levels and WBC counts were significantly elevated in patients with major amputation in the univariate analysis, these variables were not statistically significant after multivariable adjustment. In adjusted models, ABI demonstrated a borderline association with major amputation (OR 0.11, 95% CI 0.01–1.00, *p* = 0.05), suggesting a potential protective effect of preserved perfusion; however, given the wide confidence intervals, this finding should be interpreted cautiously. Patients with elevated inflammatory markers often present with more severe infection, ischemia, or comorbid conditions, which are also captured by clinical variables such as insulin therapy, reduced ABI, and overall comorbidity burden. Consequently, inflammatory markers may function more as indicators of disease severity rather than independent statistical predictors of amputation. The increased risk of major amputation among patients receiving insulin or combined therapy in our cohort likely reflects the greater disease severity and metabolic instability of advanced diabetes rather than a direct treatment effect. Advanced diabetes is frequently associated with endothelial dysfunction and reduced nitric oxide bioavailability, which contribute to microvascular impairment and delayed wound healing in diabetic foot disease [30]. Accordingly, the need for intensive insulin therapy may serve as a clinical marker of more severe metabolic and vascular dysfunction, predisposing patients to poorer limb-salvage outcomes.

Renal dysfunction has been recognized as an important determinant of poor outcomes in patients with diabetic foot disease. Reduced renal function is associated with systemic inflammation, endothelial dysfunction, and impaired immune responses, all of which may contribute to delayed wound healing and an increased risk of limb loss. Several studies have reported that decreased eGFR and dialysis dependency are associated with higher risks of major amputation in patients with diabetic foot ulcers [31, 32]. In our cohort, however, renal function assessed using eGFR was not significantly associated with either re-amputation (*p* = 0.23) or major amputation (*p* = 0.07). This finding may be related to the relatively small number of major amputation events and the limited distribution of advanced renal dysfunction in our study population. Nevertheless, renal dysfunction may influence the interpretation of inflammatory markers, as chronic kidney disease is frequently accompanied by persistent low-grade inflammation that can elevate CRP and WBC levels independent of acute infection [33]. In addition to renal impairment, systemic vascular disease may contribute to the progression of diabetic foot complications. In our cohort, higher amputation levels were accompanied by an increased prevalence of coronary artery disease (CAD). CAD has previously been reported as an important comorbidity associated with poor outcomes in patients with diabetic foot disease, as it reflects advanced systemic atherosclerosis and impaired tissue perfusion [34]. These findings suggest that amputation progression may represent not only local deterioration of foot lesions but also the clinical manifestation of systemic vascular disease. From a clinical perspective, patients with multiple severe comorbidities may also be considered less suitable for prolonged limb-salvage attempts. In such situations, clinicians may opt for a more definitive surgical strategy to avoid repeated procedures and prolonged treatment courses. Accordingly, major amputation may represent an end-stage clinical phenotype resulting from the combined effects of infection severity, vascular insufficiency, and systemic metabolic deterioration.

These findings highlight the importance of an integrated approach to diabetic foot management that considers both biological and social determinants of disease progression. Physiological factors such as metabolic instability, systemic inflammation, and vascular insufficiency directly influence wound healing and amputation risk, while social vulnerability, including the absence of spousal support, may compromise adherence to wound care and continuity of follow-up. These domains are not independent but interact synergistically, amplifying the overall risk of recurrent or higher-level amputation. Therefore, effective prevention strategies should extend beyond glycemic and wound management and incorporate multidisciplinary interventions that address metabolic control, vascular optimization, and structured psychosocial support.

Collectively, these results suggest that major amputation represents an end-stage clinical phenotype resulting from the combined failure of infection control, tissue perfusion, and metabolic regulation. Preventing these outcomes requires an integrated multidisciplinary strategy that addresses infection management, vascular optimization, and metabolic stabilization.

This study had several limitations. First, it was conducted as a single-center retrospective analysis, which may have introduced selection bias and limits the generalizability of the findings. In addition, the relatively small sample sizes of certain subgroups, particularly among patients with higher-level amputations, require cautious interpretation. Second, quantitative socioeconomic indicators such as income, educational attainment, and occupation were not available, making it difficult to fully distinguish and quantify the influence of social determinants. Third, the distribution of ulcer severity in our cohort was highly skewed, with the majority of patients (92.9%) classified as Texas grade 3 lesions. This limited our ability to adequately evaluate the prognostic impact of ulcer depth. Future studies including a more heterogeneous distribution of ulcer severity and incorporating alternative classification systems such as Wagner or SINBAD may provide a more comprehensive assessment of ulcer-related risk stratification. Fourth, osteomyelitis and the extent of surgical debridement have been reported as important predictors of revision surgery after minor amputations. However, detailed data regarding osteomyelitis severity and debridement procedures were not systematically collected in this study. Despite these limitations, this study represents one of the few single-center cohort analyses that simultaneously evaluated metabolic, inflammatory, and social support factors in patients undergoing diabetic foot amputation. Future multicenter prospective studies are needed to externally validate these findings and to develop more comprehensive predictive models incorporating clinical, imaging, and biological markers. Additionally, while marital status was identified as a significant predictor, it may primarily reflect underlying social support, and further studies incorporating direct psychosocial measures are warranted.

## Conclusion

In this single-center retrospective cohort study, both clinical and social factors were found to influence outcomes following diabetic foot amputation. Higher BMI and absence of a spouse were independently associated with an increased risk of re-amputation, while insulin or combined therapy and absence of a spouse were associated with major amputation. These findings highlight the importance of integrating metabolic, vascular, and psychosocial considerations in the management of diabetic foot disease. Future multicenter prospective studies are warranted to validate these findings and to develop more comprehensive predictive models.

## Supplementary Information

Supplementary Material 1.

Supplementary Material 2.

## Data Availability

The datasets generated and/or analyzed during the current study are not publicly available due to institutional data protection regulations but are available from the corresponding author on reasonable request.

## Abbreviations

DM: Diabetes mellitus
DFA: Diabetic foot amputation
DFU: Diabetic foot ulcer
BMI: Body mass index
CKD: Chronic kidney disease
CAD: Coronary artery disease
eGFR: Estimated glomerular filtration rate
ABI: Ankle–brachial index
CRP: C-reactive protein
WBC: White blood cell count
