# Supplementary material for: Risk factors for re-amputation and major amputation following diabetic foot amputation: a clinical and socioeconomic perspective
Source: BMC Surg. 2026 Apr 25;26:385. doi: 10.1186/s12893-026-03777-4 (PMC13251079; doi:10.1186/s12893-026-03777-4)
Supplement: Supplementary file 1 — Supplementary Material 1. [file 12893_2026_3777_MOESM1_ESM.docx]

Supplementary Table S1. Firth penalized logistic regression analysis identifying predictors of major amputation following diabetic foot amputation.

| Variable | OR | 95% CI | p-value |
| --- | --- | --- | --- |
| **Marital status¹** | 8.79 | 1.52–50.74 | **0.02** |
| **Marital status²** | 7.82 | 1.12–54.76 | **0.04** |
| Living status³ | 0.49 | 0.10–2.44 | 0.38 |
| DM treatment⁴ | 3.62 | 0.99–13.24 | 0.05 |
| ABI⁵ | 0.14 | 0.02–1.08 | 0.06 |
| CRP (mg/L) | 1.03 | 0.96–1.11 | 0.44 |
| WBC (10³/µL) | 1.05 | 0.97–1.14 | 0.23 |

Firth penalized logistic regression was performed to reduce potential small-sample bias due to the limited number of major amputation events relative to the number of predictors.

Bold values indicate statistical significance (*p* < 0.05). OR, odds ratio; CI, confidence interval

¹ Never married vs Married, ² Divorced/Widowed vs Married, ³ Living alone vs Living with family, ⁴ Insulin or combined therapy vs Oral agents, ⁵ ABI indicates ankle-brachial index; per 1-unit increase.

Reference categories for the categorical variables were Married, Living with family, Oral agents
