# Supplementary material for: Risk factors for re-amputation and major amputation following diabetic foot amputation: a clinical and socioeconomic perspective
Source: BMC Surg. 2026 Apr 25;26:385. doi: 10.1186/s12893-026-03777-4 (PMC13251079; doi:10.1186/s12893-026-03777-4)
Supplement: Supplementary file 2 — Supplementary Material 2. [file 12893_2026_3777_MOESM2_ESM.docx]

**Manuscript title:**

**Risk Factors for Re-amputation and Major Amputation Following Diabetic Foot Amputation: A Clinical and Socioeconomic Perspective**

STROBE Statement—checklist of items that should be included in reports of observational studies

| **Item No** | **Recommendation** | **Reported on Page** |
| --- | --- | --- |
| 1(a) | Indicate the study’s design in the title or abstract | Abstract, Methods |
| 1(b) | Provide an informative and balanced summary in the abstract | Abstract |
| 2 | Explain the scientific background and rationale | Introduction |
| 3 | State specific objectives and hypotheses | Introduction |
| 4 | Present key elements of study design | Methods |
| 5 | Describe setting, locations, and dates | Methods (study period: August 2024–August 2025; tertiary referral center in Korea) |
| 6(a) | Describe eligibility criteria and participant selection | Methods |
| 6(b) | Describe matching criteria (if applicable) | Not applicable |
| 7 | Define outcomes, exposures, predictors, confounders | Methods (definitions of re-amputation, major amputation, and clinical/socioeconomic predictors) |
| 8 | Data sources and measurement methods | Methods |
| 9 | Address potential sources of bias | Discussion (limitations including selection bias, small sample size, and potential residual confounding are discussed) |
| 10 | Explain how study size was determined | Methods (sample size determined based on all eligible patients during the study period) |
| 11 | Explain handling of quantitative variables | Methods (continuous variables analyzed without categorization) |
| 12(a) | Describe all statistical methods | Methods (t-test, chi-square test, multivariate logistic regression, Kaplan–Meier analysis, and Cox regression) |
| 12(b) | Methods for subgroups/interactions | Methods (no formal subgroup or interaction analyses were performed) |
| 12(c) | Handling of missing data | Methods |
| 12(d) | Handling of loss to follow-up | Not applicable (no significant loss to follow-up due to retrospective design) |
| 12(e) | Sensitivity analyses | Supplementary Table S1 |
| 13(a) | Report numbers at each study stage | Results |
| 13(b) | Reasons for non-participation | Methods (exclusion criteria described) |
| 13(c) | Flow diagram | Not applicable (study design did not require a flow diagram) |
| 14(a) | Participant characteristics | Tables 1–4 |
| 14(b) | Missing data | Methods (missing data were excluded from analyses without imputation) |
| 14(c) | Follow-up time | Results (median follow-up time and interquartile range reported) |
| 15 | Outcome data | Results (re-amputation rate, major amputation rate, and time-to-event outcomes reported) |
| 16(a) | Main results (estimates, CI) | Tables 3, 5 |
| 16(b) | Category boundaries | Methods (simplified categorization of Texas classification; definitions of minor vs major amputation) |
| 16(c) | Absolute risk (if relevant) | Not applicable |
| 17 | Other analyses (subgroup, sensitivity) | Supplementary Table S1 (Firth penalized logistic regression sensitivity analysis) |
| 18 | Summarise key results | Discussion |
| 19 | Discuss limitations | Discussion |
| 20 | Interpretation of results | Discussion |
| 21 | Generalisability | Discussion (single-center design and generalizability discussed) |
| 22 | Funding | Funding section |
